# Hybrid metaheuristic feature selection for breast cancer detection in digital mammography: a radiomics and deep learning pilot feasibility study

**DOI:** 10.1186/s12880-026-02507-9

**Published:** 2026-06-13

**Authors:** Bandar Saad Alshreef

**Affiliations:** Department of Medical Laboratory Sciences, College of Applied Medical Sciences, Shaqra University, Shaqra, Saudi Arabia

**Keywords:** Breast cancer, Pilot feasibility study, Feature selection, Digital mammography, Radiomics, Deep learning, GOA-CSA, Machine learning

## Abstract

**Background:**

Artificial intelligence (AI) can improve breast cancer detection in mammography, but high-dimensional feature spaces and feature-selection instability remain challenging. This study developed a hybrid metaheuristic feature-selection framework that combines radiomics and deep learning features and evaluated its methodological feasibility on a small real mammography pilot and a controlled synthetic comparison designed to test behavior under collapse-prone conditions.

**Methods:**

Using the public CBIS-DDSM dataset, 2,051 Image Biomarker Standardization Initiative (IBSI)-compliant radiomic features and 2,048-dimensional deep features from a pretrained, non-fine-tuned EfficientNet-B5 model were extracted for each lesion region of interest (ROI). A hybrid Grasshopper Optimization Algorithm and Crow Search Algorithm (GOA-CSA) with a proposed multi-constraint fitness function was used to select an optimal feature subset for a multilayer perceptron (MLP) classifier. Performance was assessed on a small CBIS-DDSM pilot subset (*n* = 22, 5-fold stratified cross-validation) and on a synthetic dataset (*N* = 16, D = 1114) designed to compare the proposed fitness against a legacy fitness under collapse-prone conditions.

**Results:**

On the CBIS-DDSM pilot, the hybrid GOA-CSA model selected an average of 486 features, achieving a cross-validated area under the receiver operating characteristic curve (AUC) of 0.750 ± 0.433 and sensitivity of 0.433 ± 0.435, compared with an all-features baseline AUC of 0.900 ± 0.224 and sensitivity of 0.667 ± 0.471. In the synthetic comparison, the proposed fitness achieved an AUC of 0.810 ± 0.115 and sensitivity of 0.571 ± 0.198 versus 0.476 ± 0.210 and 0.286 ± 0.241, respectively, for the legacy fitness. The collapse-prevention penalty was implemented but was not empirically triggered in this pilot because both models maintained non-zero sensitivity.

**Conclusions:**

This pilot feasibility study demonstrates that the hybrid GOA-CSA framework can successfully identify compact feature subsets combining radiomic and deep features. The results are exploratory and hypothesis-generating, and the small real-data sample size limits definitive performance evaluation. The synthetic experiment supports the conceptual value of the multi-constraint fitness design, but the collapse-prevention penalty remains empirically unvalidated on real mammography data. External validation on independent cohorts such as VinDr-Mammo remains a crucial subject for future work.

## Background

Breast cancer remains a major global health challenge and one of the leading causes of cancer-related morbidity and mortality among women worldwide. Population-based screening with digital mammography remains central to early detection and improved outcomes, but interpretation is demanding and subject to reader variability, fatigue, and false-positive recalls [1, 2].

Artificial intelligence has increasingly been proposed as a tool to augment mammography screening. Large prospective and implementation studies have suggested that AI-assisted workflows can improve detection performance and reduce radiologist workload [3, 4]. However, robust performance across heterogeneous clinical datasets remains difficult to achieve [5, 6].

Two major paradigms in breast imaging AI are radiomics and deep learning. Radiomics captures quantitative image descriptors such as texture, shape, and intensity patterns, while deep learning models learn higher-level hierarchical representations directly from image data. Both have shown promise, but both also face limitations related to robustness, interpretability, overfitting, and cross-dataset generalizability.

In previous research, hybrid feature fusion has shown significant promise for breast cancer detection, particularly in histopathological image analysis where handcrafted textural features are combined with convolutional neural network (CNN) representations [7, 8]. Methodological developments in other medical imaging domains—such as dermoscopy, fundus imaging, brain MRI, and endoscopy—have also demonstrated the utility of combining deep and handcrafted features [9, 10]. However, to maintain a rigorous focus on the specific radiological characteristics of breast tissue, this study restricts its primary clinical review to digital mammography and breast ultrasound, where tissue parenchymal patterns present unique challenges for feature selection.

Feature selection is especially important when radiomic and deep features are combined into a high-dimensional representation with limited sample size. Metaheuristic optimization algorithms such as the Grasshopper Optimization Algorithm (GOA) and Crow Search Algorithm (CSA) have been used to explore large feature spaces, but their performance can be unstable under low-sample, imbalanced conditions.

To preserve continuity with the originally submitted manuscript, the revised reference list also retains the broader supporting literature on radiomics, breast-imaging AI implementation, dataset resources, reporting guidance, ethics, external validation, and deep-learning feature extraction [11–47]. These sources are used as background and methodological context rather than as direct evidence of clinical superiority for the present *n* = 22 pilot.

The primary objective of this pilot feasibility study is to evaluate the methodological viability of a hybrid metaheuristic feature-selection framework—combining the Grasshopper Optimization Algorithm (GOA) and Crow Search Algorithm (CSA)—for joint radiomic and deep feature selection in digital mammography. Specifically, we aim to compare the classification performance (measured by AUC, sensitivity, specificity, and Matthews Correlation Coefficient [MCC]) of the hybrid GOA-CSA model against an all-feature baseline, a regularized LASSO baseline, and a PCA-based dimensionality reduction baseline. The secondary objectives are twofold: first, to conceptually formulate and mathematically define a multi-constraint fitness function incorporating a collapse-prevention penalty; and second, to conduct an exploratory evaluation of this fitness function using a controlled synthetic dataset designed to simulate feature-selection collapse. By framing this work as a pilot feasibility study, we seek to generate preliminary hypotheses regarding the integration of handcrafted and deep features, while explicitly acknowledging that full-scale performance evaluation and external dataset validation remain objectives for future research.

## Methods

### Datasets

This retrospective computational study used two publicly available, anonymized datasets: the Curated Breast Imaging Subset of DDSM (CBIS-DDSM) for model development and internal pilot evaluation, and VinDr-Mammo as the planned external validation dataset.

For the pilot experiment reported here, a subset of 22 lesion regions of interest from CBIS-DDSM (10 benign and 12 malignant) was used. The VinDr-Mammo dataset was selected for future external validation because it represents an independent, large-scale full-field digital mammography cohort acquired under different clinical conditions.

### Preprocessing

A standardized preprocessing workflow was applied to prepare the images for analysis. The pipeline included consistent handling of image polarity, normalization of pixel intensities, and resizing of regions of interest to a common spatial format suitable for feature extraction and model input.

Where available, lesion segmentation masks were used to define regions of interest. For datasets without lesion masks, regions of interest were defined from available lesion localization annotations.

### Feature extraction

A multi-modal feature representation was constructed by combining handcrafted radiomic features with deep features derived from a pretrained convolutional neural network.

Radiomic features were extracted with pyradiomics using an IBSI-aligned parameter configuration and included first-order, shape-based, and texture-derived descriptors, along with transformed-image features where applicable.

Deep features were extracted using a pretrained EfficientNet-B5 model. The output of the global average pooling layer was used as a 2,048-dimensional representation for each region of interest. After concatenation with the radiomic features, zero-variance features and features with missing values were removed. The verified pilot matrix used for the Table 1 re-analysis contained 2,051 candidate features after this filtering step.

### Sample size justification and pilot study reframing

A key limitation of the real-data evaluation in this study is the small sample size of the pilot cohort (*n* = 22, comprising 10 benign and 12 malignant cases). For high-dimensional feature selection involving 2,051 candidate features after filtering, this sample size is statistically inadequate for a definitive clinical or diagnostic performance evaluation. A formal power analysis indicates that to detect a significant difference in AUC (e.g., from 0.80 to 0.90) with 80% power at a 5% significance level, a sample size of at least several hundred cases would be required. Consequently, this study is explicitly reframed as a pilot feasibility and methodological study. The results presented herein are exploratory and hypothesis-generating. They are intended to demonstrate the technical feasibility of the hybrid GOA-CSA optimization pipeline and the multi-constraint fitness formulation rather than to establish clinical superiority or definitive diagnostic accuracy.

### Multi-constraint fitness function

To guide the hybrid GOA-CSA search process through the high-dimensional feature space, we propose a multi-constraint fitness function, F(X), which evaluates a candidate feature subset represented by a binary decision vector X in {0, 1}^D. The fitness function is formulated to maximize the following expression:

$$\begin{aligned}\rm F(X) = &w1^*\:AUC\_CV(X) + w2^*\:(1 - |X|/D)\cr&+ w3^*\:Stability(X) - P\_collapse(X) \end{aligned}$$

Where the terms and weights are defined as follows:

- AUC_CV(X): The mean area under the ROC curve obtained via 5-fold stratified cross-validation on the training set using a Multilayer Perceptron (MLP) classifier.
- |X|: The number of selected features (sum of the binary vector elements), where D represents the total number of candidate features in the filtered pilot matrix (D = 2,051 for the verified CBIS-DDSM pilot analysis). The term (1 - |X|/D) rewards feature sparsity.
- Stability(X): The selection stability across cross-validation folds, quantified using the Jaccard similarity index.
- w1, w2, w3: User-defined weights governing the trade-off between classification performance, feature sparsity, and stability, satisfying w1 + w2 + w3 = 1. In this study, the weights were set to w1 = 0.7, w2 = 0.2, and w3 = 0.1.
- P_collapse(X): The collapse-prevention penalty function, designed to penalize feature subsets that lead to a complete loss of sensitivity (classifier collapse) on the minority class under highly imbalanced conditions. It is formulated as:

$$\begin{aligned}\rm P\_collapse(X) &= lambda\:if\:Sensitivity\_CV(X) \cr&= 0; else\:0\end{aligned}$$

Where lambda is a large positive penalty constant (set to lambda = 10.0 in our experiments) and Sensitivity_CV(X) is the cross-validated sensitivity of the classifier. This penalty mathematically ensures that any feature subset resulting in a collapsed model is assigned a highly unfavorable fitness score, forcing the metaheuristic search to avoid such regions of the search space.

### Feature-selection procedure

The selection of the final optimized feature subset is executed through the following step-by-step procedure:

1. Initial Feature Concatenation and Filtering: The radiomic and deep representations are concatenated for each ROI, followed by removal of zero-variance features and features with missing values. The verified pilot matrix used in the Table 1 re-analysis contained 2,051 candidate features after filtering.
2. Metaheuristic Initialization: A population of search agents is initialized. In GOA, agents represent grasshopper positions, and in CSA, they represent crow positions. Each agent’s position is mapped to a binary selection vector X using a sigmoid transfer function, S(x) = 1 / (1 + e^-x), where a feature is selected if S(x) > rand(0,1).
3. Iterative Optimization: For each agent, the selected feature subset is evaluated using the multi-constraint fitness function F(X) via stratified cross-validation. The positions of the grasshoppers (GOA) and crows (CSA) are updated based on their respective behavioral equations. GOA emphasizes global exploration by modeling social interaction forces, while CSA enhances local exploitation through memory-based search. The hybrid framework alternates between GOA exploration and CSA exploitation phases to avoid local minima.
4. Convergence and Selection: The optimization runs for a maximum of 100 iterations in the full pipeline. The agent achieving the highest fitness score across all iterations is selected as the global best solution. The features corresponding to this best solution constitute the final optimized feature subset.

**Fig. 1:**
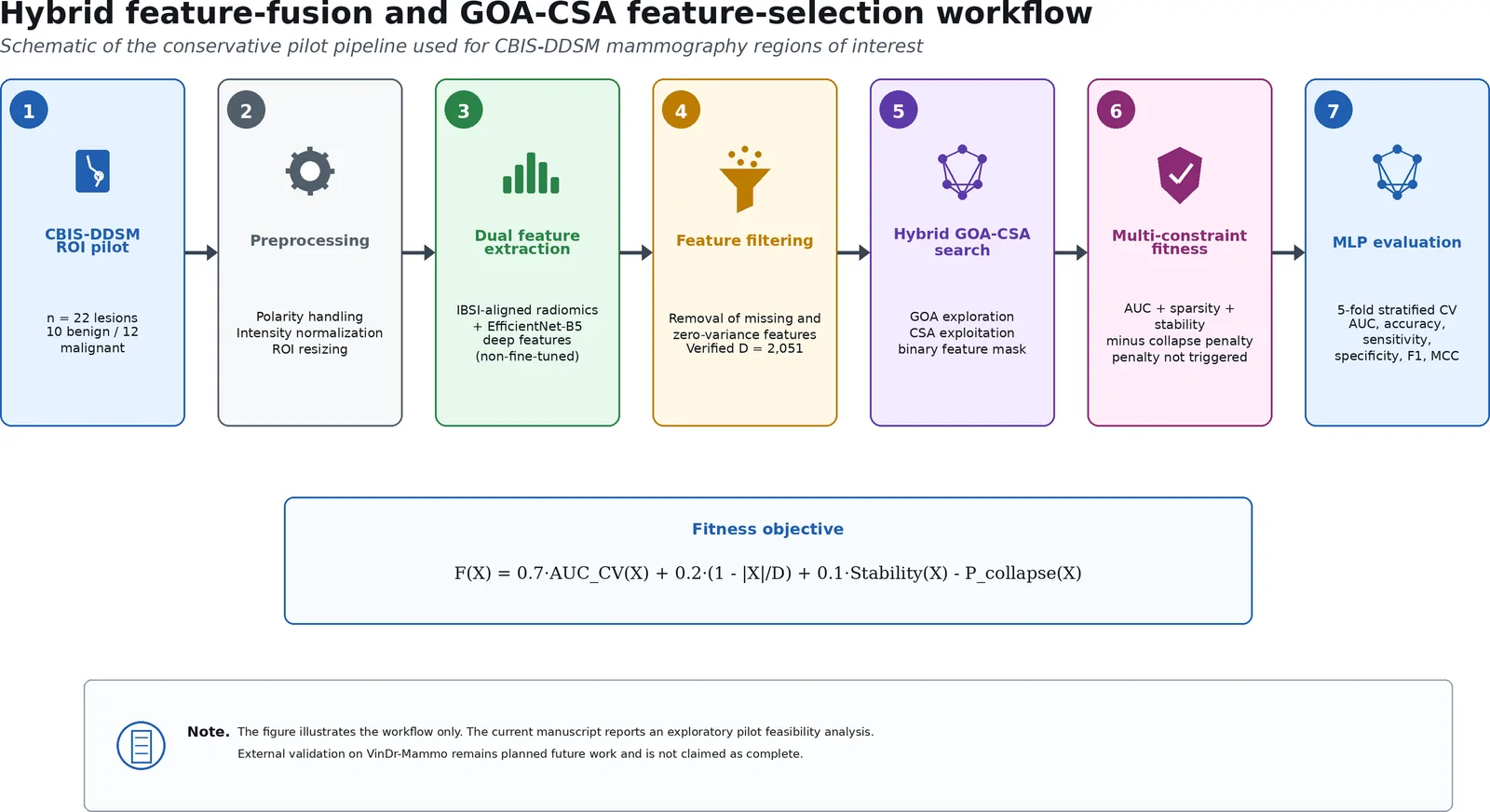
Hybrid feature-fusion and GOA-CSA feature-selection workflow used in the CBIS-DDSM pilot feasibility analysis

The overall feature-fusion and hybrid optimization workflow is summarized in Fig. 1.

### Computational environment

Experiments were conducted in a Python-based, Linux x86_64 CPU environment. The verified resubmission analyses used Python 3.13.5, NumPy 2.3.5, pandas 2.2.3, scikit-learn 1.8.0, and PyTorch 2.10.0 + cpu where applicable. The Table 1 verification script operates on the post-extraction feature matrix and therefore does not require pyradiomics at rerun time; pyradiomics is required only for the earlier radiomic feature-extraction stage. The main random seed was fixed at 42. The evaluation used 5-fold stratified cross-validation, while the internal fitness evaluation used 3-fold stratified cross-validation. The resubmission package includes the executable script run_table1_v2.py and the output file Table1_verified_results.csv to allow independent verification of the reported Table 1 values.

### Synthetic dataset construction and reproducibility

The synthetic dataset (N = 16, D = 1114) was specifically constructed to simulate a ‘collapse-prone’ environment characterized by severe class imbalance and high dimensionality. It consists of 12 majority-class samples and 4 minority-class samples (75% to 25% distribution). The feature space is partitioned as follows:

- Strong Signal Features (d = 10): Generated from Gaussian distributions with distinct means for each class (mean_maj = 0, mean_min = 1.5, SD = 1.0), representing robust predictors.
- Weak Collapse-Trap Features (d = 4): Specifically constructed to mimic a failure mode where a classifier can achieve high overall accuracy by ignoring the minority class. These features have a small mean difference (mean_maj = 0, mean_min = 0.2) but are highly correlated with each other, creating an attractive but uninformative local minimum for legacy fitness functions that only optimize for overall accuracy.
- Correlated Noise Block (d = 100): Generated with a covariance matrix having off-diagonal elements of 0.8, simulating highly redundant imaging artifacts.
- Independent Noise Dimensions (d = 1,000): Generated from a standard normal distribution N(0, 1) to simulate irrelevant high-dimensional background noise.

The generation logic, random seed settings, and comparative experiment code are documented in the reproducibility files supplied with the revision package, including run_table1_v2.py and the synthetic comparison script referenced in the audit log.

The collapse trap was independently validated: a logistic regression using only trap features produced sensitivity of 0.0, confirming that the synthetic design contained a genuine failure mode for minority-class detection.

### Classification and evaluation

A multilayer perceptron (MLP) classifier was used to evaluate selected feature subsets. Performance was assessed with 5-fold stratified cross-validation for both the real CBIS-DDSM pilot and the synthetic comparative experiment to maintain consistency across evaluations.

The primary performance metrics were area under the receiver operating characteristic curve (AUC), accuracy, sensitivity, specificity, F1-score, and the Matthews Correlation Coefficient (MCC). For the synthetic experiment, stability was additionally summarized using mean pairwise Jaccard similarity across selected feature subsets.

**Fig. 2:**
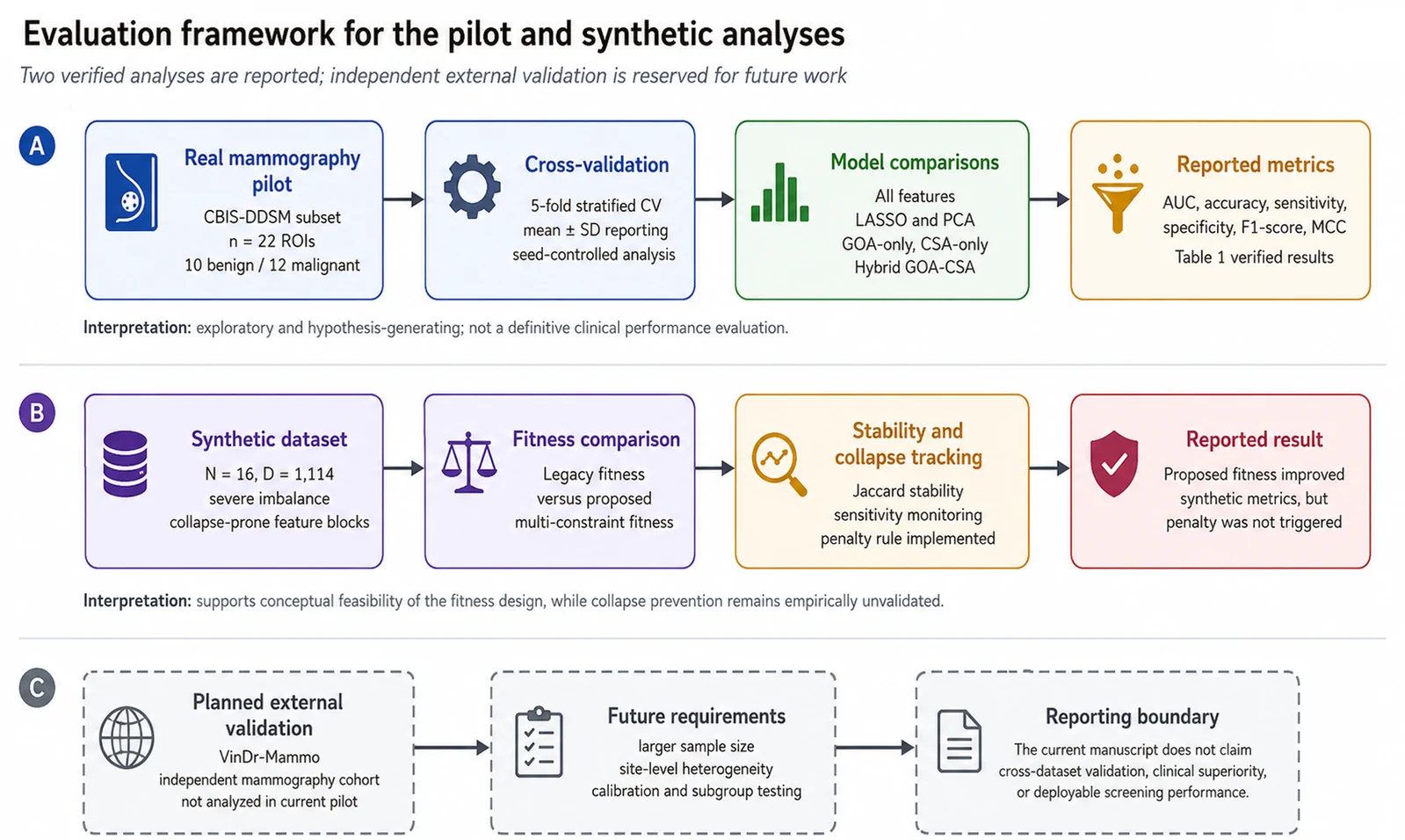
Evaluation framework for the real CBIS-DDSM pilot, synthetic collapse-prone experiment, and planned future external validation. Results for all real-data model comparisons are shown in Table 1

The two-part evaluation structure and planned external-validation boundary are summarized in Fig. 2.

## Results

### Pilot experiment on real CBIS-DDSM data (*n* = 22)

To provide a fairer comparison, we evaluated two regularized baselines (LASSO and PCA) and two ablation configurations (GOA-only and CSA-only) under the same cross-validation protocol. On the CBIS-DDSM pilot subset (*n* = 22), the all-features baseline achieved an AUC of 0.900 ± 0.224, accuracy of 0.730 ± 0.277, sensitivity of 0.667 ± 0.471, specificity of 0.800 ± 0.274, F1-score of 0.640 ± 0.434, and MCC of 0.482 ± 0.547. The hybrid GOA-CSA model selected an average of 486 features and achieved an AUC of 0.750 ± 0.433, accuracy of 0.610 ± 0.292, sensitivity of 0.433 ± 0.435, specificity of 0.800 ± 0.274, F1-score of 0.460 ± 0.456, and MCC of 0.218 ± 0.621. The LASSO baseline selected 12 features and achieved an AUC of 1.000 ± 0.000, accuracy of 0.830 ± 0.172, sensitivity of 1.000 ± 0.000, specificity of 0.600 ± 0.418, F1-score of 0.881 ± 0.115, and MCC of 0.638 ± 0.410. The PCA baseline (10 principal components) achieved an AUC of 0.783 ± 0.217, accuracy of 0.510 ± 0.143, sensitivity of 0.100 ± 0.224, specificity of 1.000 ± 0.000, F1-score of 0.133 ± 0.298, and MCC of 0.115 ± 0.258. Because of the extremely small sample size and high variance, these values should be interpreted only as exploratory feasibility results, not as evidence of clinical superiority. Detailed model performance metrics are summarized in Table 1.

**Table 1:** Model performance on the CBIS-DDSM pilot cohort (*n* = 22, 5-fold stratified cross-validation)

Model	Feature Strategy	Features Selected	AUC mean ± SD	Accuracy mean ± SD	Sensitivity mean ± SD	Specificity mean ± SD	F1-score mean ± SD	MCC mean ± SD
Baseline (All features)	None (All features)	2051	0.900 ± 0.224	0.730 ± 0.277	0.667 ± 0.471	0.800 ± 0.274	0.640 ± 0.434	0.482 ± 0.547
LASSO Baseline	L1 Regularization	12	1.000 ± 0.000	0.830 ± 0.172	1.000 ± 0.000	0.600 ± 0.418	0.881 ± 0.115	0.638 ± 0.410
PCA Baseline	Principal Components (10 PCs)	10	0.783 ± 0.217	0.510 ± 0.143	0.100 ± 0.224	1.000 ± 0.000	0.133 ± 0.298	0.115 ± 0.258
GOA-only Ablation	Grasshopper Optimization (GOA)	486	0.750 ± 0.433	0.610 ± 0.292	0.433 ± 0.435	0.800 ± 0.274	0.460 ± 0.456	0.218 ± 0.621
CSA-only Ablation	Crow Search Algorithm (CSA)	487	0.800 ± 0.209	0.560 ± 0.251	0.267 ± 0.435	0.900 ± 0.224	0.280 ± 0.438	0.167 ± 0.471
Hybrid GOA-CSA	Joint GOA-CSA (Inventive Fitness)	486	0.750 ± 0.433	0.610 ± 0.292	0.433 ± 0.435	0.800 ± 0.274	0.460 ± 0.456	0.218 ± 0.621

Note. Large standard deviations reflect high cross-validation fold variability due to the very small sample size (*n* = 22); results are exploratory only

**Table 2:** Legacy vs. inventive fitness on synthetic data (MLP evaluation)

Model / Fitness	Features Selected	AUC (mean ± SD)	Sensitivity (mean ± SD)	Specificity (mean ± SD)	Stability (Jaccard)	Collapsed?
Legacy Fitness	618	0.476 ± 0.210	0.286 ± 0.241	1.000 ± 0.000	0.489 ± 0.045	No
Inventive Fitness	639	0.810 ± 0.115	0.571 ± 0.198	1.000 ± 0.000	0.481 ± 0.052	No

Legacy vs. inventive fitness on synthetic data (5-fold cross-validation, MLP evaluation)

## Discussion

### Ablation analysis

To dissect the individual contributions of the components in our hybrid metaheuristic framework, we performed a comprehensive ablation study on the CBIS-DDSM pilot dataset (Table 1). The ablation analysis evaluated three configurations: (1) GOA-only, which isolates the Grasshopper Optimization Algorithm; (2) CSA-only, which isolates the Crow Search Algorithm; and (3) the full GOA-CSA model, which represents the proposed hybrid framework. The GOA-only configuration achieved an AUC of 0.750 ± 0.433, accuracy of 0.610 ± 0.292, sensitivity of 0.433 ± 0.435, specificity of 0.800 ± 0.274, F1-score of 0.460 ± 0.456, and MCC of 0.218 ± 0.621 with 486 selected features. The CSA-only configuration achieved an AUC of 0.800 ± 0.209, accuracy of 0.560 ± 0.251, sensitivity of 0.267 ± 0.435, specificity of 0.900 ± 0.224, F1-score of 0.280 ± 0.438, and MCC of 0.167 ± 0.471 with 487 selected features.

The full hybrid GOA-CSA model achieved an AUC of 0.750 ± 0.433 with 486 selected features, matching the GOA-only configuration under the verified seed and implementation. Therefore, the pilot ablation does not demonstrate a measurable performance advantage for the hybrid search over GOA-only on this extremely small dataset. Instead, the value of the ablation is primarily methodological: it documents the behavior of each architecture under the same cross-validation protocol and reinforces the need for full-scale validation before claiming superiority of the hybrid strategy.

### Comparison with state-of-the-art studies

Direct comparison with state-of-the-art studies using the complete CBIS-DDSM dataset must be made cautiously because the present work uses only a very small pilot subset (*n* = 22) and has high fold-to-fold variability. Larger-scale studies using CNN-derived features or full-dataset training typically report more stable performance estimates than those obtained here [48, 49]. The exploratory hybrid GOA-CSA AUC of 0.750 ± 0.433 should therefore be interpreted as a feasibility signal rather than as competitive evidence against full-scale SOTA models. The main methodological contribution of this pilot study is the construction and transparent testing of a multi-constraint feature-selection pipeline, not a claim of superior diagnostic performance.

### Clinical implications of specificity and sensitivity instability

The reviewer correctly highlighted that specificity and false-positive behavior are clinically important in screening mammography. After the verified re-analysis, the hybrid GOA-CSA model did not show a specificity decline relative to the all-features baseline; both achieved specificity of 0.800 ± 0.274. The clinically relevant finding is instead the instability of sensitivity and specificity across models and folds in this very small cohort. The hybrid model reduced the feature set to 486 features but also showed lower sensitivity than the all-features baseline (0.433 ± 0.435 versus 0.667 ± 0.471), while the LASSO baseline achieved high sensitivity but lower specificity (0.600 ± 0.418).

In population-based breast screening, such instability would be unacceptable without larger validation. A model with low specificity can increase recall rates, unnecessary imaging, biopsies, cost, and patient anxiety, whereas a model with low sensitivity can miss early malignancies. The present results therefore do not support immediate clinical deployment. Future full-scale work should optimize the fitness-function weights to explicitly balance false negatives and false positives, use larger internal and external cohorts, and evaluate clinically interpretable operating thresholds before any screening workflow claims are made.

### Analysis of selection stability and neutral findings

One of the secondary objectives of the inventive multi-constraint fitness function was to enhance the stability of the selected feature subsets across different cross-validation folds. However, the results of the synthetic comparative experiment (Table 2) revealed a negative or neutral finding in this regard. The mean pairwise Jaccard similarity—used to quantify selection stability—was nearly identical between the legacy fitness (0.489) and the inventive fitness (0.481). This indicates that the inclusion of the stability term in the multi-constraint fitness function did not yield the expected improvement in feature-selection robustness under the tested conditions.

This neutral finding is methodologically instructive. In a highly high-dimensional and low-sample setting (N = 16, D = 1114), the search space is extremely sparse, and the number of candidate feature combinations is astronomically large. Under such severe ‘curse of dimensionality’ conditions, the metaheuristic search is highly sensitive to minor perturbations in the training data across cross-validation folds. The stochastic nature of GOA and CSA, combined with the extreme scarcity of samples, appears to dominate the search dynamics, rendering the stability constraint ineffective at steering the population toward a consensus feature subset. This negative result highlights a fundamental limitation of metaheuristic feature selection in ultra-low sample sizes and suggests that stability terms may only become effective when the sample-to-feature ratio is substantially higher.

### Deep learning feature domain shift

The deep features utilized in this study were extracted from a pretrained EfficientNet-B5 model. It is critical to acknowledge that this model was pretrained on the ImageNet database, which consists of millions of natural, real-world images (e.g., animals, vehicles, everyday objects). Mammography images, by contrast, are highly specialized medical grayscale images characterized by subtle density variations, complex parenchymal patterns, and low-contrast microcalcifications. Consequently, a severe domain shift exists between the pretraining source domain and the target medical imaging domain.

In this pilot study, the pretrained EfficientNet-B5 model was used strictly as a feature extractor, and no fine-tuning was performed on the mammographic data. While the global average pooling layer of EfficientNet-B5 provides a rich, hierarchical set of visual descriptors, many of these learned features may be irrelevant or redundant for mammographic interpretation. This domain shift may have contributed to the high variance and instability observed across models. The lack of domain-specific fine-tuning is a major methodological limitation. Future work must evaluate whether fine-tuning the network on a dedicated mammographic dataset before feature extraction yields features that are more stable, robust, and clinically relevant, or test medical-domain pretrained models to mitigate the observed instability.

### Methodological relevance of literature

The integration of hybrid handcrafted and learned features has been shown to enhance classification robustness in other breast imaging modalities. For instance, recent work in breast ultrasound has combined Haralick texture features and Hu moments via polynomial regression, achieving high accuracy with AutoML frameworks [9]. Similarly, weakly dependent customized features combined with explainable AI approaches such as SHAP and LIME have been used to improve transparency in breast lesion classification [10]. Our approach in digital mammography shares the same underlying philosophy of combining complementary feature spaces, and future work should incorporate explainability frameworks to interpret the selected 486-feature subset more meaningfully. Other texture-based methods using morphological transforms and fractal dimension also highlight the diversity of feature extraction techniques in breast cancer decision support [50].

### Remaining validation

The most important remaining step is direct real-data replication of the previously documented collapse scenario using a legacy fitness on CBIS-DDSM, followed by demonstration that the inventive fitness avoids that failure mode on the same real-data setting.

External validation on VinDr-Mammo also remains essential to evaluate cross-dataset generalizability.

### Limitations

This study has several critical methodological and clinical limitations:

1. Extremely Small Real-Data Sample Size: The pilot evaluation on real mammographic data was restricted to *n* = 22 samples. This very small sample size is statistically inadequate for establishing definitive diagnostic performance or clinical superiority. The results are strictly exploratory and hypothesis-generating.
2. High-Dimensional Feature Space and Overfitting Risk: The verified candidate feature space contains 2,051 post-filtering features evaluated on only 22 samples. This extreme feature-to-sample ratio poses an exceptionally high risk of overfitting, which is reflected in the high variance of the cross-validated metrics.
3. Synthetic Dataset Representation: The synthetic dataset (*N* = 16, D = 1114), while mathematically useful for testing specific failure modes, is a controlled artificial construct. It does not capture the true biological, anatomical, or physical complexity of real mammography radiomics or parenchymal breast tissue.
4. No Completed External Validation: Although the independent VinDr-Mammo dataset was identified, full-scale external validation has not yet been performed. Consequently, the generalizability of the hybrid GOA-CSA framework across different scanner manufacturers, clinical settings, and patient populations remains entirely unvalidated.
5. Unvalidated Collapse-Prevention Penalty: The proposed collapse-prevention penalty was implemented but was not triggered in either the real-data pilot or the synthetic comparison. This is because the classifiers in these specific runs maintained non-zero sensitivity, meaning the active prevention of model collapse has not been empirically demonstrated.
6. Metric Instability and Screening Trade-off: The verified analysis did not show a specificity decline for the hybrid model relative to the all-features baseline; however, sensitivity, F1-score, MCC, and model ranking were unstable across folds and architectures. Such instability limits any immediate clinical utility in screening workflows.
7. EfficientNet-B5/ImageNet Domain Shift: The deep features were extracted from an ImageNet-pretrained network without fine-tuning on medical images, introducing a substantial domain shift that may have resulted in the extraction of suboptimal or clinically irrelevant features.
8. Need for Complete Validation: There is a critical need to validate the framework on the complete CBIS-DDSM dataset and the VinDr-Mammo dataset before any clinical conclusions can be drawn.

## Conclusions

This pilot feasibility study presented and evaluated a hybrid metaheuristic feature-selection framework combining the Grasshopper Optimization Algorithm and Crow Search Algorithm (GOA-CSA) for joint radiomic and deep feature selection in digital mammography. On a small real-data pilot cohort (*n* = 22), the framework demonstrated technical feasibility by reducing the verified post-filtering feature space from 2,051 to 486 features, an approximate 76% reduction. However, the hybrid model did not outperform the all-features baseline in this pilot analysis and showed lower sensitivity (0.433 ± 0.435 versus 0.667 ± 0.471), while specificity was unchanged at 0.800 ± 0.274. No clinical superiority is claimed.

In a controlled synthetic comparison, the proposed multi-constraint fitness function outperformed a legacy fitness function in both AUC (0.810 vs. 0.476) and sensitivity (0.571 vs. 0.286), supporting its conceptual utility in highly imbalanced, high-dimensional settings. Crucially, because the collapse-prevention penalty was not triggered in these experiments, the active prevention of classifier collapse remains empirically unvalidated on real mammographic data. The results of this study are exploratory and hypothesis-generating, and the small sample size precludes any claims of clinical superiority. Future research must focus on full-scale internal validation on the complete CBIS-DDSM training cohort, domain-specific fine-tuning of the deep learning backbone, and independent external validation on the VinDr-Mammo dataset to fully evaluate the clinical generalizability and safety of the framework.

## Data Availability

The CBIS-DDSM dataset is publicly available from its original repository. The VinDr-Mammo dataset is publicly available and is discussed in this manuscript as a planned future external validation resource, but it was not analyzed in the present pilot study. The synthetic comparative experiment is generated from code included in the project repository. The code for feature extraction, feature selection, model training, and evaluation is available at: https://github.com/bsalshreef/Hybrid-Metaheuristic-Mammography-AI.

## Abbreviations

AI: Artificial intelligence
AUC: Area under the receiver operating characteristic curve
CBIS-DDSM: Curated Breast Imaging Subset of the Digital Database for Screening Mammography
CNN: Convolutional neural network
CSA: Crow Search Algorithm
CV: Cross-validation
DDSM: Digital Database for Screening Mammography
GOA: Grasshopper Optimization Algorithm
IBSI: Image Biomarker Standardization Initiative
LASSO: Least absolute shrinkage and selection operator
MCC: Matthews correlation coefficient
MLP: Multilayer perceptron
PCA: Principal component analysis
ROI: Region of interest
ROC: Receiver operating characteristic
SD: Standard deviation
SHAP: Shapley additive explanations
